# Prescription trends of antiseizure medications before and during the COVID-19 pandemic

**DOI:** 10.3389/fneur.2023.1135962

**Published:** 2023-03-30

**Authors:** Alekhya Lavu, Donica Janzen, Laila Aboulatta, Payam Peymani, Lara Haidar, Brianne Desrochers, Silvia Alessi-Severini, Sherif Eltonsy

**Affiliations:** College of Pharmacy, University of Manitoba, Winnipeg, MB, Canada

**Keywords:** antiseizure medications, epilepsy, seizures, COVID-19, antiepileptic drugs, drug utilization, prescription patterns

## Abstract

**Introduction:**

Given the lack of evidence on how the COVID-19 pandemic impacted antiseizure medication (ASM) use, we examined the trends of ASMs before and during COVID-19.

**Methods:**

We conducted a population-based study using provincial-level health databases from Manitoba, Canada, between 1 June 2016 and 1 March 2021. We used interrupted time series autoregressive models to examine changes in the prevalence and incidence of ASM prescription rates associated with COVID-19 public health restrictions.

**Results:**

Among prevalent users, the COVID-19 pandemic led to a significant increase in new-generation ASMs with a percentage change of 0.09% (*p* = 0.03) and a significant decrease in incidence use of all ASMs with a percentage change of −4.35% (*p* = 0.04). Significant trend changes were observed in the prevalent use of new-generation ASMs (*p* = 0.04) and incidence use of all (*p* = 0.04) and new-generation ASMs (*p* = 0.02). Gabapentin and clonazepam prescriptions contributed 37% of prevalent and 54% of incident use.

**Conclusion:**

With the introduction of public health measures during COVID-19, small but significant changes in the incident and prevalent use of ASM prescriptions were observed. Further studies are needed to examine whether barriers to medication access were associated with potential deterioration in seizure control among patients.

**Conference presentation:**

The results from this study have been presented as an oral presentation at the 38th ICPE, International Society of Pharmacoepidemiology (ISPE) annual conference in Copenhagen.

## Introduction

The COVID-19 pandemic has impacted the lives of patients with chronic diseases (1, 2). Physician activity in Canada decreased by ~30–40% in April 2020 compared to 2019, and more than 25% of Canadians reported that an appointment for healthcare services was canceled, rescheduled, or delayed (3). The preventive measures to contain COVID-19 affected the living conditions of patients with chronic disease resulting in reduced physical activity, changes in diet, changes in medical care, and availability of supplies (4, 5). Concerns of COVID-19 infection, prescription medication shortages, travel and gathering restrictions, financial restrictions, and substance use are some of the reported barriers to prescription medication access among patients with chronic disease (6–11). Epilepsy is a chronic neurological disorder prevalent in ~0.7–1.0% of the population (12). While published studies have reported changes in the prescriptions for chronic diseases such as hypertension and depression during the COVID-19 pandemic, there is a lack of evidence on prescription trends of antiseizure medications (ASMs) (7, 10, 13–20). In addition to their primary use to control seizures, ASMs are used off-label to treat both acute and chronic conditions such as pain, migraine, restless legs syndrome, some psychiatric disorders, and post-traumatic stress syndrome (21). Gabapentin (GBP) and clonazepam (CLZ) are the most used medications for the off-label use of ASMs. The present study aimed to study ASM prescription trends before and during the COVID-19 pandemic in a province-wide cohort from Manitoba, Canada.

## Methods

We conducted a population-based quasi-experimental study to examine the trends of all ASM, new-generation ASM, and old-generation ASM utilization in pre-COVID-19 and during COVID-19 in Manitoba, Canada. We used the Manitoba Center for Health Policy (MCHP) repository, a provincial-level population database, to form our cohort. ASM quarterly dispensation rates were examined between 1 June 2016 and 1 March 2021, calculating the quarterly prevalence and incidence rate. As older adults have higher rates of antiseizure medication use, we dichotomized age into >65 and ≤ 65 years for descriptive results (22–26). We conducted interrupted time series analysis, using autoregressive models, to investigate changes in ASM prescriptions rates and trends before and during COVID-19, with the 2020 second quarter as the intervention point. We also calculated the relative percentage change as the relative change between the second quarter of 2020 and the first quarter of 2020 (Relative percentage change = (Percentage in Q2-2020 – Percentage in Q1-2020)/ Percentage in Q1-2020^*^100).

We examined the prescription trends of (1) all ASMs, (2) old-generation ASMs, and (3) new-generation ASMs. We classified the medications included in the analysis into the old and new generations according to previously established criteria reflecting the year of introduction to the markets, where the old generation included first-generation ASMs while the new generation included both second- and third-generation ASMs (see Supplementary Table 1 for medication list). We further conducted a subgroup analysis examining all ASMs, old-generation ASMs, and new-generation ASMs, while excluding clonazepam and gabapentin, given their increase in use for indications other than epilepsy.

## Results

**Figure 1:**
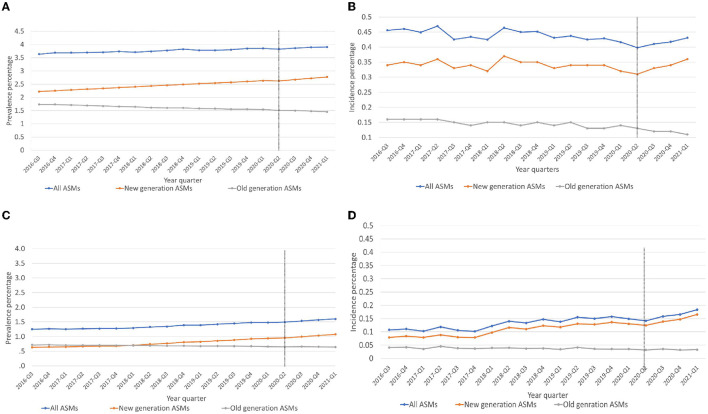
**(A–D)** Trends of prevalent and incident antiseizure medication prescriptions used. **(A)** Prevalent use of ASMs, **(B)** Incident use of ASMs. **(C)** Prevalent use of ASMs excluding Gabapentin and Clonazepam. **(D)** Incident use of ASMs excluding Gabapentin and Clonazepam, ASMs, antiseizure medications, 2020-Q2, Intervention point (COVID-19 pandemic).

We studied ~1.3 million prescriptions between 1 June 2016 to 1 March 2021, representing the incident and prevalent trends of ASM use. The population above 65 years had ASM prescriptions 2-fold higher than the < 65-year-old group, and women had a higher prevalence of ASM prescriptions than men (see Supplementary Figures 1, 2). Similar trends for age and sex were observed for incident use. During the period examined, an average of 5,709 incident ASM prescriptions/quarter and an average of 52,178 prevalent ASM prescriptions/quarter were filled. During our study period (1 June 2016 to 1 March 2021), we observed an increase in the prevalent use of new-generation ASMs and a decrease in old-generation ASM use; however, both were relatively minimal (Figure 1A). We did not observe any major changes in the incident use of all or new-generation ASMs; however, we observed a minor decrease in old-generation ASMs (Figure 1B). After excluding gabapentin and clonazepam from both incident and prevalent prescriptions, we observed a minor increase in all and new-generation ASMs and a stable rate of old-generation ASM use (Figures 1C, D).

### Prevalence

COVID-19 restrictions led to a small but significant increase in the prevalent use of new-generation ASMs by 0.09 % (*p* = 0.03). No significant change was observed among all ASMs (−0.68%, *p* = 0.12) or old-generation ASMs (−2.26%, *p* = 0.51). A significant change in prescription trends was observed in the prevalent use of new-generation ASMs (β_3_) = 0.018 (*p* = 0.04; Table 1, Figure 1).

**Table 1:** Changes in level and trends of incident and prevalent ASM prescriptions.

**ITS analysis**		**Change in level**			**Pre-COVID-19 trend**		**During COVID-19 trend**		**Change in trend**	
		**Percentage change (%)**	**Parameter estimate (**β_2_**)**	**p-value**	**Parameter estimate (**β_1_**)**	**p-value**	**Parameter estimate (**β_1_+β_3_**)**	**p-value**	**Parameter estimate** (β_3_**)**	**p-value**
**Prevalence**										
Primary objective	All ASMs	−0.68	−0.042	0.12	0.014	< 0.01	0.027	< 0.01	0.013	0.16
	New generation ASMs	0.09	−0.054	0.03	0.029	< 0.01	0.047	< 0.01	0.018	0.04
	Old generation ASMs	−2.26	0.009	0.51	−0.014	< 0.01	−0.02	< 0.01	−0.005	0.24
Secondary objective	All ASMs excluding gabapentin and clonazepam	0.69	−0.014	0.61	0.018	< 0.01	0.036	< 0.01	0.019	0.06
	New generation ASMs excluding gabapentin	1.70	−0.011	0.68	0.023	< 0.01	0.041	< 0.01	0.018	0.06
	Old generation ASMs excluding clonazepam	−0.81	−0.004	0.52	−0.004	< 0.01	−0.004	0.05	< 0.001	0.86
**Incidence**										
Primary objective	All ASMs	−4.35	−0.037	0.04	< -0.002	0.01	0.011	0.07	0.013	0.04
	New generation ASMs	−1.59	−0.035	0.06	< -0.001	0.21	0.015	0.01	0.016	0.02
	Old generation ASMs	−8.69	−0.002	0.79	−0.002	< 0.01	−0.005	0.02	−0.003	0.15
Secondary objective	All ASMs excluding gabapentin and clonazepam	−5.11	−0.029	0.02	0.004	< 0.01	0.013	< 0.01	0.009	0.03
	New generation ASMs excluding gabapentin	−4.34	−0.027	0.02	0.005	< 0.01	0.013	< 0.01	0.009	0.02
	Old generation ASMs excluding clonazepam	−9.38	−0.003	0.46	< -0.001	0.03	< 0.001	0.98	< 0.001	0.74

ASMs, antiseizure medications.

### Subgroup analysis

Excluding clonazepam and gabapentin lowered prevalent prescriptions by 37%. We observed a non-significant change in the prevalent use of all ASMs, 0.69% (*p* = 0.61), old-generation ASMs, −0.81% (*p* = 0.52), and new-generation ASMs 1.70% (*p* = 0.68; Table 1).

### Incidence

We observed a significant decrease in the incidence use of all ASMs by 4.35% (*p* = 0.04) and a non-significant decrease in new-generation ASM prescriptions by 1.59% (*p* = 0.06) and old-generation ASM prescriptions by 8.69% (*p* = 0.79). Significant trend changes in the incident prescriptions of all ASMs (β_3_) = −0.013 (*p* = 0.04) and new-generation ASMs (β_3_) = 0.016 (*p* = 0.02) were found. There was no significant trend change in the incidence use of old-generation ASM prescriptions (Table 1, Figure 1).

### Subgroup analysis

After excluding gabapentin and clonazepam, we observed a 54% decrease in the incident ASM prescriptions. We found a significant decrease in the incident prescriptions of all ASMs by 5.11% (*p* = 0.02) and new-generation ASMs by 4.34 (*p* = 0.02), while the decrease in old-generation ASM use was non-significant (−9.38%, *p* = 0.46). We observed a significant change in the incident prescription trends of all ASMs (β_3_) = 0.009 (*p* = 0.03) and new-generation ASMs (β_3_) = 0.009 (*p* = 0.02; Table 1).

## Discussion

In the current study, we found that restrictions due to the COVID-19 pandemic were associated with a small (0.09%) but significant immediate increase in new-generation ASM prescriptions among prevalent users. However, after excluding gabapentin from new-generation ASMs, there was a non-significant increase in prevalent use by 1.70%. We also found a significant 4.35% decline in all ASM incident prescriptions, and the results were consistent with a 5.11% decrease in all ASM incident prescriptions after excluding gabapentin and clonazepam. With the exclusion of gabapentin from new-generation ASMs, we found a significant decline in incident ASM prescriptions by 4.34%.

Hospital visits in Manitoba were fully suspended on March 18, reducing access to in-person care (22). Reduction in access to physician care might have had a potential impact on the diagnosis of new cases of epilepsy and short-term prescription of ASMs for non-epilepsy conditions (e.g., gabapentin or clonazepam). Therefore, these measures possibly contributed to the significant decrease in incident prescriptions of ASMs.

Different jurisdictions in Canada have reported changes in medication use during the early periods of the pandemic, with a significant increase in claims for some drugs (e.g., cardiovascular drugs and oral antidiabetic drugs) but a slight decrease in claims for controlled drugs such as opioids and benzodiazepines (16). Similar changes in chronic medication use due to the COVID-19 pandemic have been observed in other parts of the world (3, 4, 10, 12–14). For example, a study from eight European countries reported a decrease in antibiotics, COPD, and asthma medication (26). Another study among the populations of England, Scotland, and Wales reported a significant decrease in hypertensive medications due to COVID-19 (27). Moreover, Maeda et al. reported a decrease in antidiabetic medication use in Japan during the first wave of COVID-19 (28). These trends can be attributed to medication stockpiling amid fears of drug shortages and anticipated supply chain disruptions (28). We did not observe a similar pattern of stockpiling of ASMs in Manitoba, as the observed increase in new-generation ASMs was relatively small and absent among old-generation users.

Manitoba pharmacists were directed to limit dispensations of all medications to a maximum 1-month supply early in the pandemic, but pharmacies remained open as essential services throughout the pandemic (22, 29). These policies minimized the impact of COVID-19 restrictions on existing users of ASMs (i.e., prevalent users). In contrast, reduced access to in-person care and patient-reported delays in seeking care likely contributed to our observed decrease in the incident use of ASMs. However, a rapid shift to virtual care and a return of physician care activity to pre-pandemic levels by the end of the first wave may have mitigated this impact on incident prescriptions. Further studies should compare the results with other parameters of care for people with epilepsy (e.g., seizure frequency or frequency of physician contacts) before and during the pandemic.

Some limitations in our study should be acknowledged. We did not study a specific epilepsy population using a definition, since our interest was the ASM utilization and not epilepsy itself. However, by excluding clonazepam and gabapentin—the most used ASMs for non-seizure-related indications, we were able to capture a closer exclusive epilepsy population (30, 31). However, it must be noted that other ASMs may also be prescribed for non-epilepsy conditions (for example, topiramate for headaches) (32). In our study, there might be factors (e.g., age, sex, and socioeconomic status) that could act as effect modifiers for the associations observed (33). However, our sample sizes did not allow for interaction models. The study data are limited to Manitoba province in Canada from 2016 to 2021. However, the results are generalizable to similar populations of developed countries.

## Conclusion

We found small but significant changes in the prescriptions of ASMs due to the COVID-19 pandemic measures. A significant volume of prescriptions was for gabapentin and clonazepam. Further studies are needed to monitor the trend changes through the pandemic waves and examine whether such changes have had any effect at the patient level.

## Data availability statement

The original contributions presented in the study are included in the article/Supplementary material, further inquiries can be directed to the corresponding author.

## Ethics statement

The studies involving human participants were reviewed and approved by the Health Research Ethics Board (HREB) at the University of Manitoba and Manitoba Health Information Privacy Committee (HIPC). Written informed consent for participation was not required for this study in accordance with the national legislation and the institutional requirements.

## Author contributions

AL and SE: study conception, analysis, and manuscript writing. DJ, LA, PP, LH, BD, and SA-S: analysis and manuscript editing. All authors contributed to the article and approved the submitted version.
