# Supplementary material for: Prescription trends of antiseizure medications before and during the COVID-19 pandemic
Source: Front Neurol. 2023 Mar 30;14:1135962. doi: 10.3389/fneur.2023.1135962 (PMC10101333; doi:10.3389/fneur.2023.1135962)
Supplement: Supplementary file 1 [file Data_Sheet_1.DOCX]

Supplementary Material

Brief report

Alekhya Lavu, Donica Janzen, Laila Aboulatta, Payam Peymani, Lara Haidar, Brianne Desrochers, Silvia Alessi-Severini, Sherif Eltonsy*

*** Correspondence:**

**Sherif Eltonsy**

**Sherif.eltonsy@umanitoba.ca**

# Supplementary Figures and Tables

## Supplementary Figures

**Supplementary figure 1: Prescription trends of the incident and prevalent use of antiseizure medications by age**


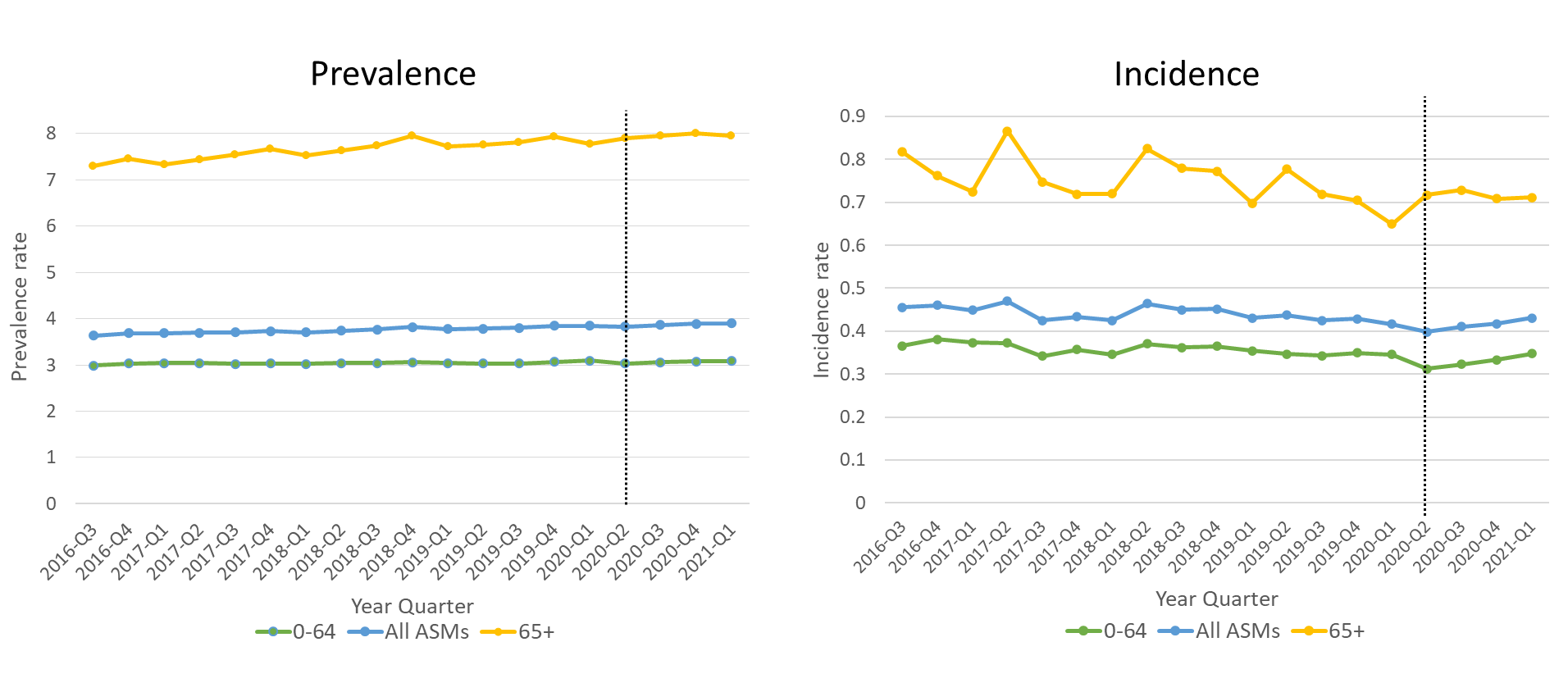


**Legend:** ASMs: antiseizure medications, 2020- Q2: Intervention point (Covid-19 pandemic)

**Supplementary figure 2: Prescription trends of the incident and prevalent use of antiseizure medications by Sex**


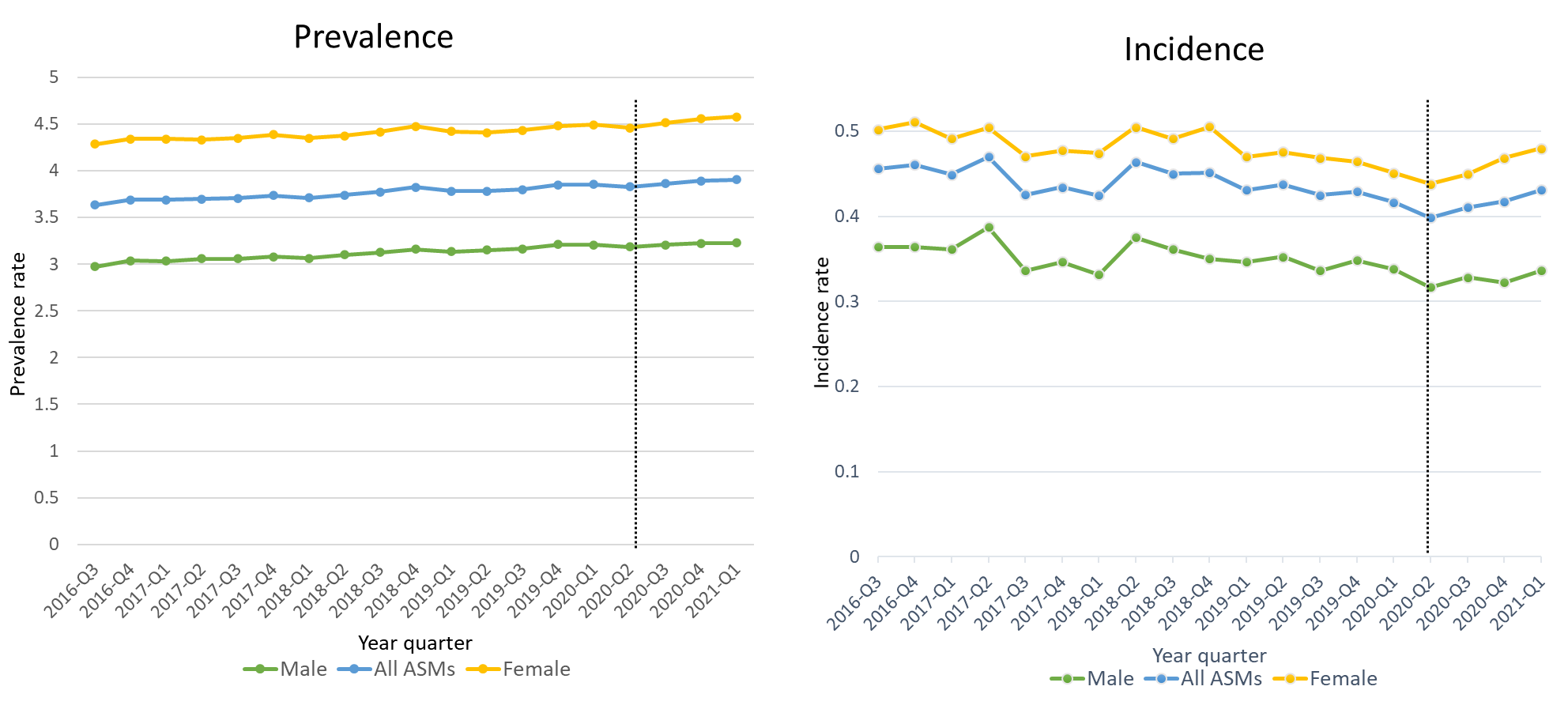


**Legend:** ASMs: antiseizure medications, 2020- Q2: Intervention point (Covid-19 pandemic)

**Supplementary Table 1**: List of antiseizure medications and their classification

| **Drug group** | **ATC code** | **Drug name** |
| --- | --- | --- |
| Any exposure | N03AF01  N03AD01  N03AD03  N03AB02  N03AA02  N03AA03  N03AG01  N03AE01  N03AX12  N03AX18  N03AX09  N03AX14  N03AF02  N03AX16  N03AF03  N03AX17  N03AX11  N03AG04 | Carbamazepine  Ethosuximide  Methsuximide  Phenytoin  Phenobarbital  Primidone  Valproic Acid  Clonazepam  Gabapentin  Lacosamide  Lamotrigine  Levetiracetam  Oxcarbazepine  Pregabalin  Rufinamide  Stiripentol  Topiramate  Vigabatrin |
| Old generation | N03AF01  N03AD01  N03AD03  N03AB02  N03AA02  N03AA03  N03AG01  N03AE01 | Carbamazepine  Ethosuximide  Methsuximide  Phenytoin  Phenobarbital  Primidone  Valproic Acid  Clonazepam |
| New generation | N03AX12  N03AX18  N03AX09  N03AX14  N03AF02  N03AX16  N03AF03  N03AX17  N03AX11  N03AG04 | Gabapentin  Lacosamide  Lamotrigine  Levetiracetam  Oxcarbazepine  Pregabalin  Rufinamide  Stiripentol  Topiramate  Vigabatrin |

.
